# Curriculum satisfaction of graduates of medical residency in ophthalmology

**DOI:** 10.1186/s12909-023-04410-1

**Published:** 2023-06-03

**Authors:** A.B.C. Guimarães, E. Amaral, K.M. Carvalho

**Affiliations:** Faculty of Medical Sciences, State University of Campinas, Rua Vital Brasil, 80. CidadeUniversitária Zeferino Vaz, Campinas, SP 13083-888 Brazil

**Keywords:** Medical education, Residency program, Curriculum, Ophthalmology

## Abstract

**Background:**

The number of ophthalmologists trained in Brazil has increased, but their satisfaction with the medical residency curriculum is unclear.

The purpose of this study is to evaluate the satisfaction and self-confidence of graduates of a reference ophthalmology residency program in Brazil and to analyze whether there is a difference in these parameters among those who graduated in different decades.

**Methods:**

This is a cross-sectional web-based study conducted in 2022 with 379 ophthalmologists who graduated from the Faculty of Medical Sciences of the State University of Campinas (UNICAMP), Brazil. We aim to obtain data on satisfaction and self-confidence in clinical and surgical practices.

**Results:**

In total, 158 questionnaires were completed (41.68% response rate); 104 respondents completed their medical residency between 2010 and 2022, 34 between 2000 and 2009, and only 20 before 2000. Most respondents were satisfied or very satisfied with their programs (98.7%). Respondents reported insufficient exposure to low vision rehabilitation (62.7%), toric intraocular implants (60.8%), refractive surgery (55.7%), and orbital trauma surgery (84.8%), particularly among graduates before 2010. They also reported insufficient training in certain non-clinical areas, such as office management (61.4%), health insurance management (88.6%), and personnel and administration skills (74.1%). We found that respondents who graduated a long time ago had higher confidence in clinical and surgical practices.

**Conclusions:**

Brazilian ophthalmology residents and UNICAMP graduates expressed high levels of satisfaction with their residency training programs. Those who completed the program a long time ago appear to have more confidence in clinical and surgical practices. There were clinical and non-clinical areas with insufficient training identified for improvement.

**Supplementary Information:**

The online version contains supplementary material available at 10.1186/s12909-023-04410-1.

## Background

Medical residency (MR) is a lato sensu postgraduate modality with the goal of medical improvement in various areas of medicine. The residency in ophthalmology used to be 2 years long, but now it has a mandatory minimum duration of 3 years in Brazil [1].

Recently, the number of ophthalmologists in the country has increased faster than the population. In 2000, there was 1 ophthalmologist for every 17,620 inhabitants in the country. The last census conducted by the Brazilian Council of Ophthalmology (BCO) in 2021 recorded 1 ophthalmologist for every 10,875 inhabitants, with a total of 19,471 ophthalmologists. Despite regional discrepancies, the number of ophthalmologists in the country is higher than what the World Health Organization recommends for developed countries (1 ophthalmologist for every 17,000 inhabitants) [2].

The observed increase could be attributed to the expansion of training opportunities, such as internships, recognized by the BCO as a proxy for the residency training, which allows these candidates to take the professional certification test at the end of 3 years of training. In 2016, 463 ophthalmologists (out of 635 enrolled) passed the National Ophthalmology Exam and received the title of specialist, granted by the BCO [3].

Therefore, the large number of ophthalmologists trained annually as well as the rejection of approximately one-quarter of candidates for the title highlight the need for minimal standardization and quality assurance of the curriculum used in residencies and internships, including the assessments conducted. It is essential to understand whether the curriculum, including skills training and the variety of clinical conditions presented to an ophthalmologist, was experienced in the residency or internship in order to be able to practice the specialty autonomously after completion of a residency program.

The three-year mandatory ophthalmology program in Brazil occurs after 6 years of medical graduation. Each program defines the number of surgeries and clinical protocols that residents must master, as long as they respect the guidelines defined by the National Residency Commission, the government entity that regulates all residency programs in Brazil. BCO has been discussing curriculum changes, including increasing the length of residency programs to 4 years and implementing core competencies [4].

In the United States, residency programs in ophthalmology already last a minimum of 4 years, with the first year dedicated to non-ophthalmic medical activities. The Accreditation Council for Graduate Medical Education (ACGME) requires more detailed performance from residents, with a minimum number of surgeries per resident. Non-technical skills such as professionalism, practice-based learning and improvement, interpersonal and communication skills, and systems-based practices are also required abilities.

In Canada, the residency in ophthalmology lasts 5 years and is guided by the Royal College of Physicians and Surgeons of Canada (RCPSC) [5].

Multiple studies have been conducted to assess the level of satisfaction with the MR program in different countries. The vast majority surveyed were residents during the program or in the first few years after its completion, with overall levels of satisfaction with the program ranging from above 90% in services in the US and below 40% among ophthalmology residents in Iran [6–11]. However, there are no studies on long-term graduates. Thus, we conducted a curriculum satisfaction survey with graduates of MR in an Ophthalmology reference program in Brazil, with training in the specialty accredited by the National Commission of Medical Residency since 1971.

## Methods

This was a cross-sectional study of physicians who have completed or are currently enrolled in the ophthalmology residency program at the Faculty of Medical Sciences of the State University of Campinas (FCM/UNICAMP). For data collection, we obtained permission to translate and adapt to the Brazilian context a questionnaire previously used in two major studies in the field [8, 9].

Since its inauguration in 1971, 398 ophthalmologists have graduated from FCM/UNICAMP. From the ophthalmology secretariat database, 379 of 398 former residents were alive and active at the time of the study. They were contacted and electronically invited to participate in the study. The project was approved by the Research Ethics Committee of UNICAMP under the number 2,194,123 and CAEE: 68159317.1.0000.5404.

In February 2022, it was sent to each former resident’s email address with a link to the form (Annex I), followed by the informed consent term (Annex II). After answering the questionnaire, the volunteer submitted the answers by clicking on a completion button, and the answers were sent to the researcher's email for analysis. There was no incentive for the participants other than their contribution to improving the residency program based on their answers and suggestions.

Two score variables were developed based on the answers of the respondents.

- Satisfaction Score: The average response to satisfaction questions, with each satisfaction question being assigned a number between 1 and 4, where 1 = very dissatisfied and 4 = very satisfied. Thus, the higher a respondent’s score, the higher their average satisfaction.
- Self-confidence Score: The average response to the self-confidence questions, with each self-confidence question assigned a number between 0 and 4, where 0 = I did not have it, 1 = no self-confidence at all, and 4 = extreme self-confidence. Thus, the higher a respondent’s score, the higher their average self-confidence. We consider a lack of training to be a sign of low self-confidence.

Residents were divided into 3 groups according to their year of MR completion: Group 1, graduated before 2000; Group 2, graduates from 2000 to 2009; and Group 3, graduates from 2010 to 2022.

To study the relationship between two categorical variables (group of the year of residency completion and procedures vs. scores), the Fisher's exact test was used, with *p* values of up to 0.05 considered significant.

## Results

The questionnaire was completed by 158 of 379 registered former residents, representing a 41.7% response rate. Most of the graduates who answered the questionnaire (65.8%) completed their MR between 2010 and 2022 (81% response rate), resulting in 104 participants, while 34 completed the residency between 2000 and 2009 (28% response rate) and 20 before 2000 (16% response rate).

Of the 158 participants who answered the questionnaire, 106 work in the state of São Paulo (66.6%) 50 in other states (31.4%), and 3 (1.9%) outside of Brazil. In terms of current activity, 91.7% reported working in clinical practice.

Thirty-nine participants (24.7%) declared being involved in academic practice, while 119 only in clinical practice. There was no significant difference in general satisfaction between the groups (Table 1).

**Table 1:** Satisfaction score depending on academic practice. (*p* = 0.091)

	**N**	**Mean**	**SD**	**IQR**	**Mininum**	**Maximum**
Academic practice	39	3.2	0.5	0.6	1.9	4
Non academic practice	119	3.1	0.5	0.8	1.6	4

*SD* Standart deviation, *IQR* interquartile range

In terms of satisfaction with the MR program, 156 participants (98.7%) reported being satisfied or very satisfied. Most were satisfied or very satisfied with classroom teachings, operating rooms, outpatient care settings, hospital visits, meetings to discuss morbidity/mortality causes in the service, videos of surgical procedures, lectures, and article discussions.

The vast majority of participants responded that they were satisfied or very satisfied with the volume (94.9%), complexity (96.8%), and variety (96.2%) of surgical cases they encountered during their MR (Table 2a). In addition, 88% thought the feedback received in the outpatient clinic was adequate or extremely adequate and advantageous, similar to the 79.8% satisfaction rate with the feedback in the operating room.

**Table 2:** Levels of satisfaction and surgical volume achieved by respondents

	**Very satisfied**	**Satisfied**	**Unsatisfied**	**Very unsatisfied**
**a) Operative experience**				
**Volume**	82 (51.9%)	68 (43.0%)	5 (3.2%)	3 (1.9%)
**Complexity**	99 (62.6%)	54 (34.2%)	2 (1.3%)	3 (1.9%)
**Variety**	95 (60.1%)	57 (36.1%)	3 (1.9%)	3 (1.9%)
**b) Quality of teaching**				
**Didact**	61 (38.6%)	85 (53.8%)	12 (7.6%)	0 (0%)
**Operating room**	71 (44.9%)	69 (43.7%)	17 (1.8%)	1 (0.6%)
**Clinic**	72 (45.6%)	72 (45.6%)	13 (8.2%)	1 (0.6%)
**Hospital rounds**	40 (25.3%)	73 (46.2%)	36 (22.8%)	9 (5.7%)
**Grand Rounds**	48 (30.4%)	74 (46.8%)	29 (18.4%)	7 (4.4%)
**Conferences**	40 (25.3%)	65 (41.1%)	43 (27.2%)	10 (6.3%)
**Wet lab**	21 (13.3%)	51 (32.3%)	66 (41.8%)	20 (12.7%)
**Number of procedures**		**N (%)**		
**c) Surgical volume**				
Cataract				
1–100		47 (29.7%)		
101–200		58 (36.7%)		
201–300		27 (17.1%)		
301–400		5 (3.2%)		
> 400		7 (4.4%)		
CR		14 (8.9%)		
Glaucoma surgery (trabeculectomy and tube implantation)				
0		46 (29.1%)		
1–5		48 (30.4%)		
6–10		18 (11.4%)		
11–20		22 (13.9%)		
21–50		17 (10.8%)		
> 50		2 (1.3%)		
CR		5 (3.2%)		
Squint (Strabismus surgery)				
0		24 (15.2%)		
1–5		63 (39.9%)		
6–10		30 (19.0%)		
11–20		15 (9.5%)		
21–50		14 (8.9%)		
> 50		5 (3.2%)		
CR		7 (4.4%)		

*N/A* not answered

Regarding the quality of the teaching received in MR, most of them were satisfied or very satisfied in the scenarios of formal didactic teaching, operating room, outpatient clinic, hospital visits, grand rounds, meetings to discuss causes of morbidity/mortality in the service, videos of surgical procedures, lectures and discussions of articles. Nevertheless, the majority declared themselves dissatisfied or very dissatisfied with scenarios of Wet lab, surgical and virtual surgical simulation system (Table 2b).

Most participants reported being dissatisfied or very dissatisfied with their learning in the surgical wet lab and surgical virtual simulation system scenarios, and less than half reported feeling self-confident or extremely self-confident in performing refractive surgery and implanting refractive toric intraocular lenses. The findings were focused on groups I and II (older residences).

Regarding cataract surgeries, 61.4% declared they have performed over 100 surgeries. However, only 26% declared they have performed over 10 surgeries of glaucoma (trabeculectomy and tube implantation) and strabismus (Table 2c).

Regarding the evaluation (ophthalmological examination) of children, 90.5% reported feeling self-confident or extremely self-confident. In terms of managing children with strabismus, this percentage dropped to 64.6%. Only 37.3% reported being self-confident or extremely self-confident in the prescription of low vision rehabilitation therapies and optical aids.

In Table 3, we compiled the responses of the participants regarding their level of confidence in performing ophthalmological procedures.

**Table 3:** Self-confidence in performing ophthalmological procedures

	**Extremely self-confident**	**Self-confident**	**Not self-confident**	**Not self-confident at all**	**I received no training**
	N (%)	N (%)	N (%)	N (%)	N (%)
**Prescribe glasses**	115 (72.8)	43 (27.2)	0 (0)	0 (0)	0 (0)
**Prescribe contact lenses**	64 (40.5)	66 (41.8)	20 (12.7)	7 (4.4)	1 (0.6)
**Perform phacoemulsification**	62 (39.2)	57 (36.1)	25 (15.8)	9 (5.7)	5 (3.2)
**Perform extracapsular cataract extraction**	65 (40.5)	59 (37.3)	24 (15.2)	8 (5.1%)	3 (1.9)
**Implant toric intraocular lens**	37 (23.4)	25 (15.8)	36 (22.8)	26 (16.5)	34 (21.5)
**Perform refractive surgery**	31 (19.6)	39 (24.7)	39 (24.7)	22 (13.9)	27 (17.1)
**Perform corneal surgeries**	22 (13.3)	44 (27.8)	49 (31.0)	26 (16.5)	18 (11.4)
**Perform laser trabeculoplasty**	22 (13.9)	15 (9.5)	28 (17.7)	24 (15.2)	69 (43.7)
**Manage surgical complications of glaucoma**	20 (12.7)	42 (26.5)	37 (23.4)	29 (18.4)	30 (19.0)
**Treat eyelid trauma**	39 (24.7)	78 (49.4)	31 (19.6)	7 (4.4)	3 (1.9)
**Treat orbit trauma**	9 (5.7)	15 (9.5)	44 (27.8)	39 (24.7)	51 (32.3)
**Perform eyelid surgery**	32 (20.4)	64 (40.5)	45 (28.5)	14 (8.9)	43(1.9)
**Perform lacrimal duct surgery**	7 (4.4)	18 (11.4)	55 (34.8)	65 (41.1)	13 (8.2)
**Perform enucleation**	75 (47.5)	63 (39.9)	16 (10.1)	4 (2.5)	0 (0)
**Evaluation of children**	59 (37.3)	84 (53.2)	14 (8.9)	1 (0.6)	0 (0)
**Manage children with strabismus**	29 (18.4)	73 (46.2)	42 (26.6)	14 (8.9)	0 (0)
**Low vision rehabilitation**	19 (12.0)	40 (25.3)	49 (31.0)	44 (27.8)	6 (3.8)

Most respondents rated some aspects of non-clinical skills in ophthalmological practice as little or inadequate, particularly aspects of management and proficiency in information technology (Table 4).

**Table 4:** Comfort level with non-clinical skills

	**Extremely suitable**	**Suitable**	**Less suitable**	**Not suitable**	**I had no skill**
**Professionalism**	65 (41.1%)	84 (53.2%)	8 (5.1%)	1 (0.6%)	0 (0.0%)
**Management skills**	18 (11.4%)	43 (27.2%)	49 (31.0%)	10 (6.3%)	38 (24.1%)
**Interpersonal and communication skills**	40 (25.3%)	72 (45.6%)	24 (15.2%)	5 (3.2%)	17 (10.8%)
**Medical knowledge**	79 (50.0%)	71 (44.9%)	4 (2.5%)	1 (0.6%)	3 (1.9%)
**Practice with medical insurance**	7 (4.4%)	11 (7.0%)	46 (29.1%)	30 (19.0%)	64 (40.5%)
**Relationship with other health professionals**	39 (24.7%)	86 (54.4%)	16 (10.1%)	9 (5.7%)	8 (5.1%)
**Personnel management and administration**	10 (6.3%)	31 (19.6%)	45 (28.5%)	21 (13.3%)	51 (32.3%)
**Information technology**	9 (5.7%)	31 (19.6%)	41 (25.9%)	20 (12.7%)	57 (36.1%)
**Relationship with physicians of other specialties**	40 (25.3%)	79 (50.0%)	23 (14.6%)	10 (6.3%)	7 (3.8%)

There was a significant difference between the groups, with residents who graduated after 2010 having a lower average self-confidence score (*p* = 0.001) (Table 5).

**Table 5:** Level of self-confidence depending on the year of graduation. (*p* = 0.001)

	**N**	**Mean**	**SD**	**IQR**	**Mininum**	**Maximum**
**Before 2000**	20	2.7	0.5	0.6	1.7	3.5
**2000–2009**	34	2.6	0.5	0.6	1.7	4
**2010–2022**	104	2.3	0.5	0.7	0.8	3.5

*SD* Standart deviation, *IQR* interquartile range

When the satisfaction score variable was examined, no significant difference was found between the 3 groups (*p* = 0.067) (Table 6).

**Table 6:** Level of satisfaction depending on the year of graduation (*p* = 0.067)

	**n**	**Mean**	**SD**	**IQR**	**Mininum**	**Maximum**
**Before 2000**	20	3.2	0.5	0.6	2.4	4
**2000–2009**	34	3.2	0.5	0.9	2.3	4
**2010–2022**	104	3	0.5	0.7	1.6	4

*SD* Standart deviation, *IQR* interquartile range

## Discussion

This study evaluated physicians who completed their ophthalmology residency at FCM-UNICAMP between 1971 and 2022. About two-thirds of the participants in this study work as ophthalmologists in the state of São Paulo, where they also completed their MR. Studies show that MR is the most relevant factor in settling a physician, which may explain the high proportion of them working in Campinas [12, 13].

### Satisfaction with medical residency program

General satisfaction with the MR program was found to be quite high. A similar rate (93.6%) was found in a study with North Americans [6]. Another study involving recent ophthalmology residency graduates in Brazil found that 89.1% were satisfied or extremely satisfied with the acquisition of clinical knowledge, 93.4% with the acquisition of surgical skills, and 74.9% with the development of the doctor–patient relationship [7]. In a study of Canadian residents and newly graduated ophthalmologists, Zhou et al. found an 85% satisfaction level. [8] However, Mostafei et al. found only a 36.4% satisfaction rate among ophthalmology residents in Iran [9].

In the Canadian study mentioned above, despite the high level of satisfaction, approximately 35% reported having received adequate clinical training in all subspecialties of ophthalmology [8]. Newly graduated ophthalmologists in India expressed a strong desire for additional training and reported little confidence in practicing ophthalmology independently [14, 15]. A study of ophthalmologists trained between 2006 and 2011 in Jordan found a 62.3% dissatisfaction level with residency programs [10]. Residents and newly graduated ophthalmologists in Croatia completed a questionnaire about their residency curriculum, including the number of procedures performed, and 79% said that they were dissatisfied [11].

If an MR is required to make the specialist competent in all areas of activity, these findings highlight the importance of individually evaluating all essential contents and competencies and reflecting on the need for criterion-based evaluation without pitting one against the other in competence-based education. The difficulties in assessing the competencies expected in medical training prompted the Competency-Based Medical Education (CBME) movement and the rediscovery of Entrustable Professional Activities as well as learning and assessment milestones to follow the development of residents' competencies with their specific skills and knowledge [16].

Some studies have shown that increasing resident satisfaction with the residency program may improve resident performance and productivity [17, 18]. Our study found no statistically significant difference between the year of completion of MR and the satisfaction score.

### Self-confidence with ophthalmic activity

The amount of knowledge and procedures that resident physicians must learn is increasing. This is reflected in the training time and the self-confidence of new specialists. A study of ophthalmologists trained less than 5 years ago in the US showed that nearly two-thirds felt the need for additional surgical training and approximately half for additional clinical training [19]. Some of the surgeries that we included in the data collection, such as the use of toric lenses and refractive surgery, were only introduced in the 1990s. Graduates from the previous period as well as those from a few years later did not have the necessary experience during their MR training.

We found a significantly higher level of self-confidence in ophthalmologists who graduated a long time ago. One possible explanation is that the younger ones are inexperienced and will require additional training in subspecialties.

Cataract surgery is one of the most common procedures in ophthalmic practice, and much of the surgical training in MR focuses on this surgery and its complications. In terms of the surgical volume of cataract surgeries, 61.4% performed more than 100 procedures during their MR, which is comparable to North American residents who performed an average of 113 phacoemulsification cataract surgeries [20]. To meet an acceptable standard, an ophthalmology resident must perform at least 86 cataract surgeries, according to the *Accreditation Council for Graduate Medical Education*, the body that accredits and evaluates medical residencies in the United States [5].

Despite this, 29.7% of respondents said they had performed fewer than 100 cataract surgeries, which is considerably higher than the 5% reported by Canadian residents [8]. This is because our sample included ophthalmologists from earlier times when there were structural and equipment limitations.

Our study found that 75.3% of the respondents felt confident or extremely confident in performing cataract surgery using the phacoemulsification technique, which is similar to that found among Canadian residents (85%) [8]. Refractive surgery had much lower rates; only 44.3% of the residents reported that they felt self-confident or extremely self-confident, compared to 10% of the Canadian residents.

### Non-clinical competencies

Non-clinical or non-technical competencies are described as fundamental for a subspecialist, ophthalmologist, or other physician to be able to fully exercise their medical practice [21–23]. Physicians face leadership challenges in routine patient care that could be better addressed with a broader skill set [24].

McDoneell et al. studied recently graduated ophthalmologists in the US and found a large difference in self-evaluation of clinical vs. non-clinical practice. Seven of the ten non-clinical areas studied had extremely negative self-evaluations, with a focus on finance, coding, and reimbursement. More than a quarter believed they needed to learn more about administrative procedures. [19] Zhou et al. found that 65% of the respondents reported having no training in administrative skills. [8] Similarly, in our study, respondents reported not having received training or receiving inadequate training (more than 50% responding) in the following areas: management skills, practice with medical insurance, personnel management, administration, and information technology.

Greig et al. found that United Kingdom curricula in postgraduate training had deficiencies on learning objectives or assessment recommendation for non-clinical skills such as leadership, decision-making, team-working and resource management [25].

Dubois et al. conducted a study with radiotherapy professionals of the Greater Region, including residents. They found that more than a third of the surveyed population recommended to further emphasize non-technical skills in their education. Communication and team training were areas highlighted as requiring more training from a professional perspective [26].

Non-medical skills were addressed as areas of scarce competency among residents. Networking of universities and hospitals with private practice would contribute to learn the basics of practice management and administrative skills, identified as a need by the respondents. We should also improve training in some areas: supervised Low Vision experiences and multidisciplinary training in places that specialize in elderly care, which would be important for improvement of the skill set in the area of low vision rehabilitation. Since most hospitals that host MR programs in ophthalmology do not perform Refractive surgeries, education on it could be ameliorated with internships in specialized private clinics.

Li et al. demonstrated that simulation modules can be used in non-clinical areas of medical training such as finance and teamwork. The study was performed on residents from four different areas, including ophthalmologists, and received positive feedback from those undergoing the simulation. There was no discernible difference in feedback among participants from different specialties [27].

### Establishing a medical curriculum in a large Country

With the number and distribution of training programs in Brazil, clinical experiences may vary according to the program [28, 29]. Thus, ophthalmology residency accreditation systems must consider local epidemiology and program profiles as well as validate the different programs within their characteristics. The importance of context in accreditation, licensing, or certification processes is a current topic under discussion in the relevant literature [30]. As a result, the BCO, as an accrediting body for residency programs, should consider an essential program for all, while also defining activities and experiences that distinguish professional training programs.

Brazil is a country of continental dimensions, presenting significant cultural and socio-economic variations among and within its regions, as well as variable complexity of the health care offered through its mixed system, public and private. The increase of ophthalmology residency services, in the last years, predominantly associated with private clinics, even if previously approved by the National Residency Commission, allowed some services to be created without adequate structure.

We believe that establishing essential competencies and entrustable professional activities is essential. However, services of different regions could also have some autonomy over the curriculum, in order to consider the regional epidemiology as well as the best experiences they can offer.

### Limitations

There are some important limitations to this study. The survey response rate was 41.7%. However, this is in line with other similar medical education survey response rates [7–9]. Most respondents completed medical residency after 2010, likely due to more accurate contact information, introducing the likelihood of self-selection bias.

One of the great challenges of this study was to include participants who graduated a long time ago; it took some effort to contact older graduates. We believe that this response rate, although asymmetrical, allows us to clarify some evidence among ophthalmology residents.

Socioeconomic and general performance characteristics in the BCO specialist licensing tests are similar between respondents and non-respondents.

Only two of the interviewees, who belonged to the 2010–2022 cohorts, referred considering their MR programs unsatisfactory experiences. We also considered the possibility of self-selection bias, since some ex-residents that considered their programs unsatisfactory might have chosen not to answer.

Regarding the questionnaire used in this study, we opted to use this instrument lacking a formal validation because there was no other option already validated. We considered its content to be valid since it involves technical information relevant to the specialty and since all respondents are specializing or have already completed their MR in this area. We opted for having a certified translator and the use of the questionnaire was authorized by its author (Dr. Nizar Abdelfattah).

Finally, although one of the largest and most traditional ophthalmology residency departments in the country, the results are from a single program.

## Conclusions

An ophthalmology resident’s training must be adjusted in response to technological advances and epidemiological demands. The need for the detailed analysis and adequacy of the medical curriculum becomes essential as the volume of knowledge grows. We found high satisfaction levels among former residents of a reference program in ophthalmology in Brazil that was comparable to American and Canadian services. We found that ophthalmologists who were trained a long time ago had higher levels of self-confidence in performing clinical and surgical procedures. One possible explanation would be the inexperience of the younger ophthalmologists and the probable need for more training in subspecialties. Efforts should be made for curricular and scenarios improvement to offer opportunities for training in the clinical and non-clinical areas identified for improvement.

## Supplementary Information

**Additional file 1.**

**Additional file 2.**

**Additional file 3.**

## Data Availability

A “xlsx” file (RESEARCH DATA.xlsx) with all data was attached. All data generated or analysed during this study are included in the supplementary information files.

## Abbreviations

FCM/UNICAMP: Faculty of Medical Sciences, State University of Campinas
MR: Medical residency
BCO: Brazilian Council of Ophthalmology
CBME: Competency-Based Medical Education
