# Supplementary material for: Curriculum satisfaction of graduates of medical residency in ophthalmology
Source: BMC Med Educ. 2023 Jun 3;23:403. doi: 10.1186/s12909-023-04410-1 (PMC10239158; doi:10.1186/s12909-023-04410-1)
Supplement: Supplementary file 1 — Additional file 1. [file 12909_2023_4410_MOESM1_ESM.pdf]

Curriculum satisfaction of graduates of medical residency in ophthalmology

You are being invited to participate as a volunteer in a survey.

This study will gather information from ophthalmologists trained in medical residency at Unicamp. The objective is to assess satisfaction with teaching and training in clinical and surgical subareas. In addition, we aimed to compare the levels of satisfaction with those of residents from other countries and compare the number of surgical procedures performed during the residency with international recommendations.

The questions will be about your perceptions of your residency program and the assessments you have participated in. The answers will be carried out in a single step.

Completing the survey form below assumes acceptance of the free and informed consent form available at
h ttps://drive.google.com/file/d/1vJ6Ht6aUKKS3mLFTlTrkr39tJHUHUIn9/view? u sp=sharing

Thanks for participating!

1. In what year did you complete your residency? *

2. What activity are you currently carrying out? (You can tick more than one option)

- Intern in Brazil
- Intern outside Brazil
- Working in clinical practice
- Seeking an internship in Brazil
- Seeking an internship outside Brazil
- Working in academic practice

3. In which city and state are you operating? Ex: Rio de Janeiro/RJ

4. What is your level of overall satisfaction with your residency program at Unicamp?

- Very Satisfied
- Satisfied
- Dissatisfied
- Very Dissatisfied

5. How do you feel about your surgical experience in the following areas?

(Very Satisfied; Satisfied; Dissatisfied; Very Dissatisfied)

- Volume of cases
- Complexity of the cases
- Variety of cases

6. How do you feel about the quality of teaching in the scenarios below?

(Very Satisfied; Satisfied; Dissatisfied; Very Dissatisfied)

- Formal didactic teaching
- Ambulatoriial surgical room
- Visit hospitals
- (patients interned e..e..)
- Grand rounds (Meeting for
- case discussions)
- Meeting to discuss the causes of morbidity/death of the service
- Wett llab surgical
- Videos of surgical procedures
- Surgical virtual simulation system
- Discussion of articles

7. How many cataract surgeries have you performed during your residency?

8. How many cataract surgeries, specifically by the technique of Phacoemulsification, did you perform it during your residency?

9. How many trabs/tube implants have you had during your residence?

10. How many strabismus surgeries did you perform during your residency?

11. How safe do you feel to perform the following procedures cataract/refraction?
(Extremely self-confident; Self-confident; Not self-confident; Not self-confident at all; I received no training)

- Prescribe glasses
- Prescribe contact lenses
- Perform phacoemulsification
- Perform extracapsular cataract extraction
- Implant toric intraocular lens
- Perform refractive surgery
- Perform corneal surger

12. How safe do you feel to perform the following glaucoma procedures?
(Extremely self-confident; Self-confident; Not self-confident; Not self-confident at all; I received no training)

- TREC/SLT
- Glaucoma complications surgery

13. How confident do you feel about performing the following retinal procedures?
(Extremely self-confident; Self-confident; Not self-confident; Not self-confident at all; I received no training)

- I fell confidente to perform posterior segment examination
- I fell confidente to perform vitreo-retinal surgical procedures

14. How safe do you feel to perform the following oculoplastic procedures?
(Extremely self-confident; Self-confident; Not self-confident; Not self-confident at all; I received no training)

- Treat eyelid trauma
- Treat orbit trauma
- Perform eyelid surgery
- Perform lacrimal duct surgery
- Perform enucleation
- Treat conjunctival tumors

15. How safe do you feel to perform the following pediatric procedures?
(Extremely self-confident; Self-confident; Not self-confident; Not self-confident at all; I received no training)

- Evaluation of children
- Manage children with strabismus
- Low vision rehabilitation

16. With regard to the following non-clinical competencies of ophthalmic practice did you receive adequate training during your residency?
(Extremely self-confident; Self-confident; Not self-confident; Not self-confident at all; I received no training)

- Professionalism
- Management skills
- Interpersonal and communication skills
- Medical knowledge
- Practice with medical insurance
- Relationship with other health professionals
- Personnel management and administration
- Information technology
- Relationship with physicians of other specialties
