# Supplementary material for: Curriculum satisfaction of graduates of medical residency in ophthalmology
Source: BMC Med Educ. 2023 Jun 3;23:403. doi: 10.1186/s12909-023-04410-1 (PMC10239158; doi:10.1186/s12909-023-04410-1)
Supplement: Supplementary file 2 — Additional file 2. [file 12909_2023_4410_MOESM2_ESM.pdf]

INFORMED CONSENT TERM

Curriculum satisfaction of graduates of medical residency in ophthalmology

Author: Aron Barbosa Caixeta Guimarães

CAAE number: 68159317.1.0000.5404

You are being invited to participate as a volunteer in a survey. This document, called the Free and Informed Consent Form, aims to ensure your rights as a participant and is prepared in two copies, one that you should keep and the other for the researcher.

Please read carefully and calmly, taking the opportunity to clarify your doubts. If there are questions before or even after signing it, you can clarify them with the researcher. If you prefer, you can take this Agreement home and consult with your family members or others before deciding to participate. There will be no penalty or loss of any kind if you do not accept to participate or withdraw your authorization at any time.

Justification and objectives:

There is little national information on the curricular composition and on the satisfaction and technical achievement during the ophthalmology residency in Brazil.

This study will gather information from newly graduated ophthalmologists in medical residency with the aim of assessing, on a national scale, the perception of ophthalmologists who completed their residency a few years ago regarding satisfaction with teaching and training in clinical and surgical subareas. In addition, we aimed to compare the levels of satisfaction with those of residents from other countries and compare the number of surgical procedures performed during the residency with international recommendations.

Procedures:

By participating in the study, you are being invited to answer an online form. The questions will be about your perceptions of your residency program and the assessments you have participated in. The answers will be carried out in a single step.

Discomforts and risks:
The discomforts and risks related to your participation are not foreseeable.

Benefits:

The benefits of the research are to know the collective perceptions about training in ophthalmology in medical residencies in the national territory and to assess whether the curriculum is compatible with that recommended by international institutions.

Follow-up and assistance:

After the end of the research, participants will receive an email with the results and conclusions obtained from the study, maintaining the absolute confidentiality of the participants and institutions to which they belong.

Confidentiality and privacy:

You have the guarantee that your identity will be kept confidential and no information will be given to other people who are not part of the research team. In disclosing the results of this study, your name will not be mentioned.

Reimbursement and Indemnity:

There will be no reimbursement of any expenses for accessing the form. You will be guaranteed the right to compensation for any damages resulting from the research.

If at any time the research participant feels uncomfortable for any reason, choosing to interrupt their participation and withdrawing consent will not cause any harm to them.

Contact:
If you have any questions about the research, you can contact the researcher:

Dr. Aron Barbosa Caixeta Guimarães

Rua Vital Brasil, 251 - Cidade Universitária "Zeferino Vaz" | Campinas - SP - Brasil - CEP 13083-888

Ophtalmology Department – HC-UNICAMP

55 (19) 98424.6268

e-mail: aronbcg@gmail.com

In case of denouncements or complaints about your participation and about ethical issues of the study, you can contact the secretariat of the Research Ethics Committee (CEP) of UNICAMP from 08:30 am to 11:30 am and from 1:00 pm to 5:00 pm :00hs at Rua: Tessália Vieira de Camargo, 126; CEP 13083-887 Campinas – SP; phone (19) 3521-8936 or (19) 3521-7187; email: cep@fcm.unicamp.br.

The Research Ethics Committee (CEP).

CEP's role is to assess and monitor the ethical aspects of all research involving human beings. The National Research Ethics Committee (CONEP) aims to develop regulations on the protection of human beings involved in research. Plays a coordinating role in the network of Research Ethics Committees (CEPs) of the institutions, in addition to assuming the role of a consulting body in the area of research ethics

Informed consent:

After receiving explanations about the nature of the research, its objectives, methods, anticipated benefits, potential risks and the inconvenience it may cause, I agree to participate:
Name____
Signature____

Researcher Responsibilities:

I certify that I have complied with the requirements of resolution 466/2012 CNS/MS and complementary ones in the elaboration of the protocol and in obtaining this Free and Informed Consent Form. I also assure that I explained and provided a copy of this document to the participant. I inform you that the study was approved by the CEP before which the project was presented. I promise to use the material and data obtained in this research exclusively for the purposes foreseen in this document or according to the consent given by the participant.
